# The primary care practitioner and the diagnosis of occupational diseases

**DOI:** 10.1186/1471-2458-10-405

**Published:** 2010-07-09

**Authors:** Luca Cegolon, John H Lange, Giuseppe Mastrangelo

**Affiliations:** 1 Padua University, Department of Environmental Medicine and Public Health, Padua, Italy; 2 Imperial College London, School of Public Health, St Mary's Campus, Norfolk Place, London, UK; 3 Envirosafe Training and Consultants, Pittsburgh, Pennsylvania, USA

## Abstract

**Background:**

Rather than a clinical diagnosis, in occupational medicine the critical point is the etiological diagnosis. The first is useful for the therapy, the latter for preventive, epidemiological, regulatory, and insurance measures.

**Discussion:**

As with causality criteria which are employed in population studies, the answering of four easy questions allows a Primary Care Practitioner to establish a causal link between the work activities and a potential disease that a specific patient may present.

After determining the clinical diagnosis and the actual pathology of an occupational disease, the identity, duration, and intensity of the exposure have to be detected for establishing a close-causal effect. The judgment on the occupational origin of the disease requires an integrated approach using multiple sources of information, and goes beyond the clinical diagnosis. This may require consultation with a specialist in occupational medicine.

**Summary:**

It is important that the Primary Care Practitioner takes an accurate medical history since this may be the only chance a patient has to have their occupational disease recognised and properly detected/identified. Proper identification of the causative nature of such diseases is important for establishing preventive measures in eliminating and controlling future cases against exposure, epidemiological reporting and studies (particularly in identifying the rates of disease), regulatory reporting requirements and insurance compensation.

## Background

A historical judgment by the Venice Appeal Court (Italy) in 2004 finally put an end to a controversial class-action law suit, which began over 10 years prior, by denouncing the deaths of 120 workers by various types of tumours. These workers had been employed since the 1950s in the chemical plants at Porto Marghera (Italy) and were involved in the production of vinyl chloride (VC) and polyvinyl chloride (PVC). VC and PVC were recognised cancer agents by the International Agency for Research on Cancer (IARC) and the European Economic Area (EEA) since the 1970s and repeated reports, notifications and denouncements about their possible role in the health problems of several workers in the 70s and 80s at Porto Marghera fell on deaf ears and were disregarded. Despite the occupational situation at these industrial plants being defined as "alarming" by some experts since 1977, governmental insurance companies were not willing to recognise the potential occupational nature of the health problems affecting the blue-collar workers, thus continuously denying them compensation. On 22 August 1994 the prosecuting attorney decided to intervene and began by investigating the real occupational mortality and disease rates of the workers involved in the management of VC and PCV, as data provided thus far by the industrial companies, underestimating the phenomenon, were not convincing [1]. On the assumption that the results of any consultations undertaken by occupational doctors employed by the industrial companies at this time were probably biased, the most reliable and effective health care resource available to these workers would have probably been their Primary Care Practitioner (PCP).

In occupational medicine the critical point is not clinical diagnosis (e.g. rhinitis), but instead the etiological diagnosis (e.g. rhinitis caused by anhydrides). The first diagnosis is useful for therapy while the latter is utilised for preventive, epidemiological, regulatory and insurance measures.

It has been reported that PCPs continue to do a poor job of obtaining an occupational history and it has been identified that additional medical education is needed to correct these inadequate practices [2,3]. These issues are of particular importance since PCPs are often the first to see patients with occupational diseases [4]. This makes their findings of critical importance since they will often make the initial determination and sometimes undertake the only evaluation as to whether the patient's condition is a result of an occupational event.

## Discussion

### Setting up an hypothesis of work

To detect an occupational disease it is necessary to investigate some aspects which are often neglected by solely taking the patient's medical history. The clinical history could be supplemented by employing some questions which rely on some of the criteria for cause-effect relationship normally used in occupational epidemiology population studies [5]. The answering of only four easy questions could be sufficient for a PCP to suspect a causal link between the work activities and the disease of a specific patient [3,6].

The suspicion of an occupational disease is reasonable when the cause precedes the effect of an interval sufficient for the development of the pathological process (query 1) and one or more positive answers are obtained from the queries 2-4 of table 1[5-7].

**Table 1:** The four questions giving rise to suspicion of the occupational nature of a disease.

HISTORY TAKING IN OCCUPATIONAL MEDICINE	
***Criteria for causal relationship in epidemiology***	***Queries to be addressed to the patient***

Temporal relationship	1. What is the time lag between the initial exposure and the start of the symptoms ?
Dose-effect relationship	2. Do the symptoms improve if the patient is not exposed any longer (e.g. if he/she changes work duties or is on holiday? 3. Do the symptoms worsen if the patient carries out specific duties or works in areas characterised by high levels of exposure?
Strength of the association	4. Are colleagues affected by the same symptoms related to the same exposure?

### Expounding upon the information collected

It is necessary to define with clinical diagnosis the actual pathology of a disease along with the identity, duration and intensity of the exposure agent. This can be elucidated by identifying a specific chemical substance, physical, biological or psychological risk factor for that patient [8-10]. A simple inquiry about the work duties of the patient does not provide adequate information and in some cases this alone may be misinterpreted by the PCP [11].

Establishing the existence of an occupational disease often requires the examination of:

1 Evidence from epidemiological studies where the disease in question is recognised as caused by the same exposure agent (substance);

2 Chemical data sheets, reporting the chemical substances contained in each product; in particular information such as "risk sentences", describing the types of symptoms potentially caused by the substance;

3 List of occupational diseases related to that specific industry or agricultural activity/practice;

4 List of mandatory notifiable diseases.

In the case of occupational diseases the physician must [12-14]:

1 Notify the occurrence of this disease case to the legal/regulatory authorities, if there is any legal responsibility for such notification;

2 Notify the occurrence of the occupational disease case to the public health authorities (if it is mandatorily notifiable);

3 Certify the occupational disease so the patient can file/claim compensation from the government/insurance companies for this disease state.

### Problems and solutions

Determining the duration of the exposure can be difficult and in some cases frustrating (particularly if there have been repeated periods of exposure and the patient has moved in and out of exposure scenarios), but should not cause great difficulty for the physician [6]. Simply identifying the type or types of exposure that the patient has from their employment can be accomplished through use of common occupational medicine/toxicological/industrial hygiene textbooks [15-17]. By contrast, deriving the intensity of exposure from the patients' employment history can be far more complicated [6,18]. However, the simple answer is that if the worker used/uses Personal Protection Equipment (PPE) (masks/respirators, gloves, etc.) this may be sufficient to provide a basic answer that exposure is likely to be occurring; although, may not provide sufficient information as to whether the PPE is sufficiently protective. In fact there are different forms of PPE and each has varying value in providing protection against harmful agents [6,19-21]. For instance, gloves protect against solvents and other toxicants only if chosen *with the awareness of the type of materials suitable in each case*[19].

To fully understand potential exposure, it is sometimes necessary to obtain the complete occupational history of the patient, occasionally going back several decades, as illustrated with the diseases mesothelioma and liver angiosarconoma. These diseases can occur up to 40 years since the exposure to asbestos [22] and vinyl chloride (VC) [23] respectively. This will assist the PCP in assessing the workplace exposure, preventing misunderstandings of practices and activities in the workplace and their over-reliance on descriptive information provided by the patient [11].

Finally, the judgment on the occupational origin of the disease can require an integrated approach using multiple sources of information. For instance, the presentation of cough and dyspnoea in a worker who smokes does not necessarily signify that the symptoms are solely attributable to smoking [24].

The guidelines hereby mentioned should be used as a preliminary tool. For further investigation on the potential occupational nature of a disease physicians should subsequently refer to the online database of the National Library of Medicine's Toxicology and Environmental Health Information Program (TEHIP). TEHIP is a relevant portal reporting extensive toxicological information worldwide, including several scientifically peer-reviewed databases residing within the web-based TOXNET system [25]. Haz-Map is one of these occupational toxicology databases available on the National Library of Medicine's TOXNET system. Haz-Map is designed primarily for health and safety professionals, but also for consumers seeking information about the health effects of exposure to chemicals and biological compounds at work. It links jobs and hazardous tasks with occupational diseases and their symptoms. The 1,595 chemicals and biological agents in the database are related to industrial processes and other activities such as hobbies. The linkage indicates the potential for exposure to the agents. The 224 occupational diseases and their symptoms are associated with hazardous job tasks. This association indicates an increased risk for significant exposure and subsequent disease [26]

As the clinical diagnosis of the disease may require appropriate tests (laboratory tests, diagnostic imaging) the etiological diagnosis may need consultation from a specialist in occupational medicine for adequate confirmation.

Lastly it is important for the PCP to be vigilant and aware that patients/workers might also be reluctant to disclose information on their potential occupational exposure because of the repercussions this may have on other people and their employer.

## Summary

The taking of an accurate and complete history by the PCP may sometimes be the only chance a patient has to have the occupational nature of his/her disease detected [4,27]. Failure in this respect can result in the patient not being listed in the appropriate registry for the relevant disease, the patient being denied insurance/governmental benefits and furthermore, the opportunity to take preventative measures in an attempt to eliminate future cases in other workers may be missed [28,29].

Taken together, these factors will prevent the case from being included in epidemiological data, thus resulting in an underestimation or miscount of the true number of cases of a specific occupational disease [30].

A simple guide, as presented here, will assist PCPs overcome any of their inadequacies in occupational medicine, especially related to their understanding of the workplace environment [11], with the ultimate aim being to achieve a higher rate of preventable disease along with a mechanism for reducing occupational compensation for these diseases [22]. It will also allow physicians to gain a better understanding of occupational events and workplace illnesses through self-education involving case-by-case evaluation of occupational diseases. In addition we hope that by educating PCPs in this way their failure rate in detecting occupational disease will be reduced and thereby increase the surveillance of specific diseases by a resultant increase in the reporting by PCPs to regulatory agencies and, in part, to the epidemiological literature [30].

For these reasons it is crucial that the PCP be alert and aware of the complexities involved in this type of diagnosis and thoroughly investigates and pursues all avenues of inquiry into the disease(s) of their patients. Overall, PCPs are often the gatekeepers for detection, diagnosis and treatment of occupationally related diseases, which places them in a unique position to provide adequate care to these patients [4] along with obtaining occupational compensation for the injured worker [11]. Inadequate evaluation will only hinder the future prevention and proper identification and reporting of occupational diseases. Undertaking comprehensive evaluations into the patient's disease can have a dramatic impact on the patient's total health and well-being and result in improved quality of life [22].

## Abbreviations

**VC**: Vinyl Chloride; **PVC**: Polyvinyl **C**hloride; **PCP**: Primary Care Practitioner; **IARC**: International Agency for Research on Cancer; **EEA**: European Economic Area; **PPE**: Personal Protection Equipment; **TEHIP**: Toxicology and Environmental Health Information Program.

## Competing interests

The authors declare that they have no competing interests.

## Authors' contributions

LC conceived the idea and developed the paper. JHL contributed in the drafting of the paper. GM conceived the idea and is the guarantor. All authors read and approved the final manuscript

## Pre-publication history

The pre-publication history for this paper can be accessed here:

http://www.biomedcentral.com/1471-2458/10/405/prepub
